# Manipulation of Molecular Spin State on Surfaces Studied by Scanning Tunneling Microscopy

**DOI:** 10.3390/nano10122393

**Published:** 2020-11-30

**Authors:** Zhen Xu, Jing Liu, Shimin Hou, Yongfeng Wang

**Affiliations:** 1 Key Laboratory for the Physics and Chemistry of Nanodevices and Center for Carbon-Based Electronics, Department of Electronics, Peking University, Beijing 100871, China; zhenxu@pku.edu.cn; 2 Division of Quantum State of Matter, Beijing Academy of Quantum Information Sciences, Beijing 100193, China

**Keywords:** molecular spintronics, spin state manipulation, scanning tunneling microscopy and spectroscopy, Kondo effect, spin excitation, spin crossover, metal phthalocyanines, lanthanide complexes

## Abstract

The adsorbed magnetic molecules with tunable spin states have drawn wide attention for their immense potential in the emerging fields of molecular spintronics and quantum computing. One of the key issues toward their application is the efficient controlling of their spin state. This review briefly summarizes the recent progress in the field of molecular spin state manipulation on surfaces. We focus on the molecular spins originated from the unpaired electrons of which the Kondo effect and spin excitation can be detected by scanning tunneling microscopy and spectroscopy (STM and STS). Studies of the molecular spin-carriers in three categories are overviewed, i.e., the ones solely composed of main group elements, the ones comprising 3d-metals, and the ones comprising 4f-metals. Several frequently used strategies for tuning molecular spin state are exemplified, including chemical reactions, reversible atomic/molecular chemisorption, and STM-tip manipulations. The summary of the successful case studies of molecular spin state manipulation may not only facilitate the fundamental understanding of molecular magnetism and spintronics but also inspire the design of the molecule-based spintronic devices and materials.

## 1. Introduction

Magnetic molecules have attracted extensive interest during the past few decades due to their potential applications as high-density memories, displays, sensors, and so forth [1,2,3,4]. Recently, magnetic molecules are drawing even more attention, since they are considered as a promising material platform for the blossoming fields of molecular spintronics and quantum computing, in which the spin degree of freedom plays the key role [5,6,7,8,9,10,11,12,13,14]. One of the crucial issues toward the development of magnetic-molecule-based devices and functional materials is the spin state manipulation of the molecular spin-carriers. The relevant studies focusing on the surface-confined molecular systems and at molecular level are of special significance not only for the fundamental understanding of the magnetic characteristics of the molecular systems but also for realizing their practical applications.

The investigation of the molecular spins absorbed on surfaces has been facilitated by the rapid development of surface science techniques over the past few decades, especially scanning tunneling microscopy (STM) whose ultra-high spatial resolution in real space makes it one of the most powerful tools for directly charactering the surface-confined molecular systems at angstrom scale [15,16,17,18,19]. The detection of magnetic properties of molecules on surfaces with STM is enabled by measuring the differential conductance (d*I*/d*V*) spectra of the molecules, which refers to scanning tunneling spectroscopy (STS). Specifically, for the magnetic molecular systems absorbed on surfaces, Kondo resonance and spin-excitation-induced inelastic electron tunneling are frequently observed by STS measurement, and they are taken as the key evidence for analyzing the spin information of the systems, such as spin state and magnetic interactions [20,21,22,23,24,25,26,27,28,29,30]. Kondo resonance, which appears as the spectral anomaly near the Fermi level, is originated from the exchange interaction between the localized spin of magnetic molecules and the conduction electrons of the substrate [31]. Thus, the Kondo effect is usually observed in the systems where there is relatively strong interaction between the molecular spin-carrier and the conducting substrate. Inelastic electron tunneling can be induced by the spin excitation of the magnetic molecules with split spin states due to external magnetic field or spin-orbit interaction [28,32,33]. It is usually observed as step-like features that are symmetric with respect to the Fermi level in the d*I*/d*V* spectra.

This review focuses on the manipulation of the electron spins of surface-confined molecules, aiming at overviewing the recent progress achieved by molecular-level investigations based on STM and STS. The main body of the review is divided into three parts, each of which summarizes the studies addressing one of the categories of magnetic molecules, i.e., the molecules solely composed of main group elements, the 3d-metal complexes, and the 4f-metal complexes. The examples presented show the application of various methods for controlling the spin state of the molecular systems, including chemical reactions, reversible atomic/molecular chemisorption, and STM-tip manipulations.

## 2. Spin State Manipulation of Molecules Composed of Main Group Elements

### 2.1. Metal-Free Molecules

Ranging from small inorganic radicals such as NO [34], open-shell graphene nanostructures [35,36,37,38,39,40,41,42,43], to pure organic spin carriers [44,45,46,47,48,49,50,51,52], metal-free molecules make up an important family of molecular magnetic materials. According to the different origins of the electron spins in the molecules, the metal-free molecular spin-carriers that have been scrutinized on surfaces can be divided into two categories: the open-shell radical molecules [34,35,36,38,39,40,41,42,43,44,45,46] and the molecules involved in significant charge transfer with either the substrate [47] or the neighboring molecules [48]. The unpaired electrons in these metal-free molecules usually reside in π molecular orbitals. Controlled generation or quenching of the magnetic moment has been achieved in several on-surface metal-free molecular systems.

**Figure 1:**
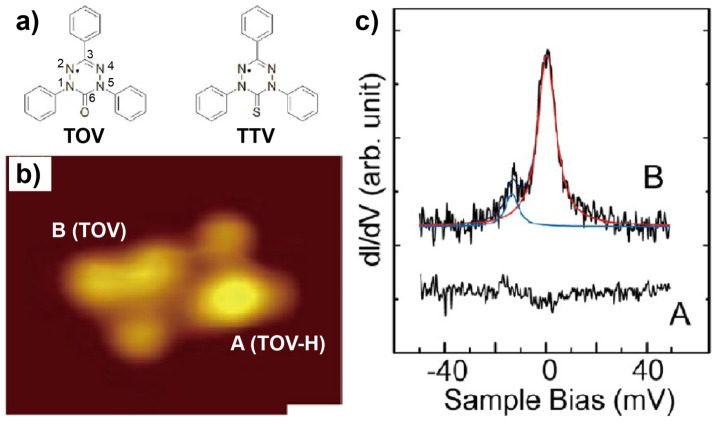
(**a**) Chemical structures of 1,3,5-triphenyl-6-oxoverdazyl (TOV) and 1,3,5-triphenyl-6-thioxoverdazyl (TTV). (**b**) Scanning tunneling microscopy (STM) image of the TOV molecules of types A and B on Au(111). (**c**) Differential conductance (d*I*/d*V*) spectra recorded at TOV molecules of types A and B. Adapted from [44] with permission from The American Chemical Society, 2013.

Liu and coworkers reported one of the earliest works concerning the controlled quenching of spins carried by the pure organic radicals absorbed on surfaces [44]. Their combined STM and density functional theory (DFT) investigations were focused on the verdazyl radicals (Figure 1a), that is, a family of pure organic molecules with an unpaired π-electron delocalized over the N(1)-N(2)-C(3)-N(4)-N(5) portion of the heterocycle, that were deposited on Au(111). They found that some 1,3,5-triphenyl-6-oxoverdazyl (TOV) molecules (denoted as type B in Figure 1b) could preserve their spin upon adsorption on Au(111), which was evidenced by the Kondo resonance seen in their d*I*/d*V* spectra (top curve in Figure 1c). As a comparison, other TOV molecules that possessed a protrusion in the center (denoted as type A in Figure 1b) showed no Kondo feature by d*I*/d*V* measurement (bottom curve in Figure 1c). Moreover, featureless d*I*/d*V* spectra were also found for another verdazyl radical, 1,3,5-triphenyl-6-thioxoverdazyl (TTV, Figure 1a). The absence of Kondo effect in the TOV molecules of type A and TTV was explained as a result of the quenched molecular spin. For the TOV molecules with a protruding center (type A), the variation in the molecular morphology compared with that of type B was attributed to the attachment of a H atom to the oxygen atom of TOV. The addition of atomic H caused the transfer of an additional electron to the verdazyl ring and thus quenched the unpaired electron spin of TOV. As for the TTV molecules absorbed on Au(111), the spin quenching was originated from the charge transfer facilitated by the strong interaction between the S atom in the molecule and the Au substrate. Therefore, it can be concluded that the spin of the TOV radical absorbed on Au(111) could be quenched by either the addition of atomic H or chemical modification of the molecule by replaying the O atom with a S atom.

**Figure 2:**
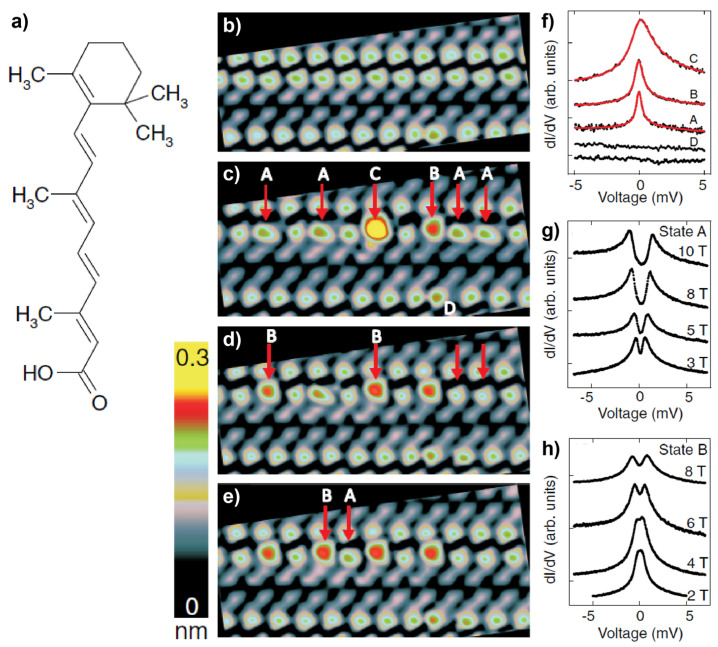
(**a**) Chemical structure of retinoic acid (ReA). (**b**) STM image of the striped pattern formed by ReA molecules in their pristine form as deposited on Au(111). (**c**–**e**) STM images of the same area after manipulating the marked molecules at a sample voltage *V* = −2.5  V. (**f**) d*I*/d*V* spectra recorded at pristine (bottom curve) and four manipulated states of ReA. Magnetic field evolutions of states (**g**) A and (**h**) B. Adapted from [54], with permission from American Physical Society, 2016.

The above-mentioned work by Liu et al. [44] shows the relatively poor stability of the pure organic spin systems adsorbed on surfaces, which is an obstacle to their characterization. In this context, controlled chemical reactions have been employed as an efficient approach to creating stable organic spins from closed-shell molecules on surfaces [53,54,55]. One of the successful cases was reported by Karan and coworkers [54]. The authors managed to convert a closed-shell all-*trans*-retinoic acid (ReA) molecule (Figure 2a) on Au(111) into a radical that carries localized spin via intramolecular chemical conversions induced by the biased STM tip. As shown in Figure 2b, ReA molecules were found to form a striped pattern on Au(111) upon adsorption. By placing the tip at a bias of −2.5 V over the neck of the cyclohexene head, the ReA molecules in arrays could be switched selectively among different states of A, B, and C which featured distinct shapes and apparent heights in the STM images (Figure 2c–e). DFT calculations enabled the authors to assign the pristine and switched molecular states to different adsorption configurations. Further spectroscopic investigations revealed the different spin states between the pristine and switched states of ReA. The d*I*/d*V* spectra of molecules in states A, B, and C displayed a remarkable peak around the Fermi level which was absent for the pristine molecules (Figure 2f). The splitting of the zero-bias peaks in the presence of a magnetic field (Figure 2g,h) demonstrated that the zero-bias peaks were originated from the Kondo effect. The latter served as a key proof for the existence of a majority spin residing in the switched molecules, although the pristine ReA is a closed-shell molecule. The follow-up theoretical study [56] attributed the emergence of spin upon molecular switching to the migration and dissociation of the allylic hydrogen from the endocyclic bond, i.e., a sigmatropic reaction, induced by the negatively biased tip. The distorted molecular structures of states A, B, and C, as well as the weak coupling between the molecules and the inert Au(111) substrate were believed to be important for stabilizing the metastable radical states of ReA. The generation of spins in organic systems by controlled on-surface chemical reactions were also reported for cholesterol molecules [53] and 13-*cis*-retinoic acid [55] on Au(111).

### 2.2. Main-Group-Metal Phthalocyanines

In addition to the metal-free molecules, some main-group-metal-involved metal–organic complexes, such as main-group-metal phthalocyanine (Pc) molecules, are also of interest in terms of molecular magnetism. Pc, a widely used macrocyclic chelating ligand, possesses four indole rings bridged by four –N= groups with their two –NH– and two –N= groups pointing toward the center of the molecule. The coordination of Pc with metal atoms usually takes place via the dissociation of the two H atoms in the –NH– groups of the indole rings, leading to a –2 valence state of the coordinated Pc ligand. Therefore, the stable metal phthalocyanine (MPc) molecules prefer to form between Pc and the metals that can be stabilized in their +2 oxidation state, such as the 3d-metals. As a comparison, for the main-group metals that not tend to stabilize in their +2 state, e.g., K (+1), Al (+3), and Ti (+4), the synthesis of the Pc-coordinated complexes and the following characterization and manipulation of their spin-related properties are challenging.

**Figure 3:**
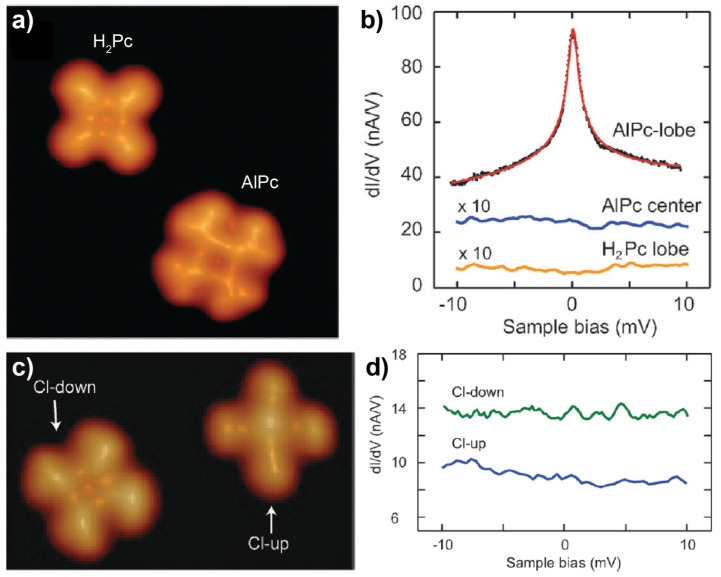
(**a**) STM image of H_2_Pc (top-left) and AlPc (bottom-right) on Au(111). (**b**) d*I*/d*V* spectra of H_2_Pc and AlPc. (**c**) STM image of ClAlPc. (**d**) d*I*/d*V* spectra of ClAlPc in either a Cl-up or a Cl-down configuration. Adapted from [57], with permission from the Royal Society of Chemistry, 2016. Pc: phthalocyanine.

Hong et al. [57] employed the recently developed vacuum synthesis method for the preparation of AlPc, a main-group-metal phthalocyanine molecule, via the metalation of pristine H_2_Pc on Au(111). The formation of the new species was confirmed by its distinct appearance in STM images (Figure 3a) and the different electronic structures as detected by d*I*/d*V* measurements compared with H_2_Pc. The existence of a spin in AlPc was evidenced by the detection of a zero-bias peak in the d*I*/d*V* spectrum acquired at the lobe of AlPc (top-most curve in Figure 3b) which was assigned to Kondo resonance according to its splitting in the increasing magnetic field. The theoretical results provided insights into the origin of the spin by showing a doublet ground state of AlPc where the singly occupied molecular orbital (SOMO) and singly unoccupied molecular orbital (SUMO) were both in a π* state. Therefore, it is concluded that the spin density of the molecule was originated from the unpaired π-electron, which was mainly delocalized over the lobes of the Pc ring. This explained the absence of Kondo resonance at the center of AlPc (middle curve in Figure 3b). Further comparative investigation of chlorinated AlPc (ClAlPc), an AlPc derivative formed by the axial bonding of the Al atom with a Cl atom, on Au(111) (Figure 3c) showed no Kondo signature for the absorbed ClAlPc molecules (Figure 3d), since all molecular orbitals of ClAlPc became doubly occupied upon chlorination. As a consequence, the spin state of AlPc on Au(111) could be tuned by the attachment/detachment of a Cl atom to the Al center.

## 3. Spin State Manipulation of Molecules Comprising 3d-Metals

### 3.1. 3d-Metal Phthalocyanines

MPcs are one of the most studied molecular systems on surfaces [58,59], among which 3d-MPcs draw special attention due to their diversified magnetic properties. With the different electronic configurations of their 3d orbitals and thus the existence of unpaired d-electrons, the central metal ions in 3d-MPcs can serve as spin-carriers presenting various spin states. Extensive investigations addressing the magnetism of 3d-MPcs confined on surfaces, such as the ones with M = V [60,61], Mn [62,63,64,65], Fe [21,23,26,28,66,67], Co [20,68], Ni [69], and Cu [69], have been reported. Various spin-state manipulation strategies have been developed for the on-surface 3d-MPc systems. In the following, we will describe by examples the two frequently used strategies for tuning the spin state of 3d-MPc molecules on surfaces: tip-induced chemical reactions and reversible atomic/molecular chemisorption.

**Figure 4:**
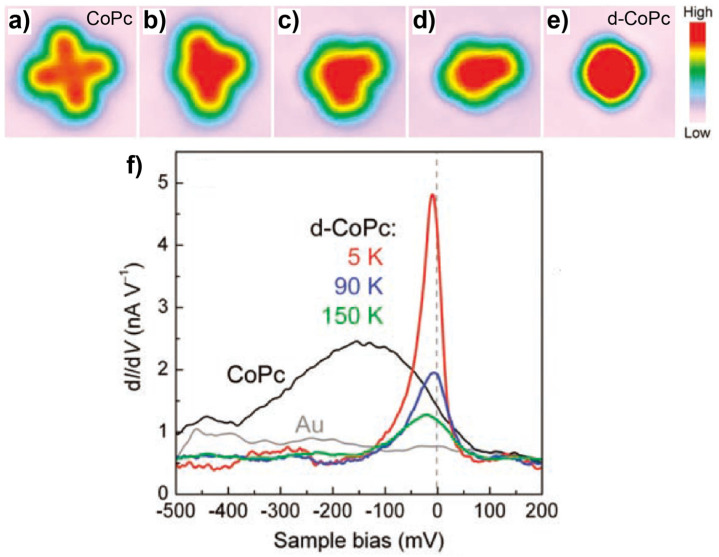
(**a**–**e**) STM images showing the sequential tip-induced dehydrogenation of a CoPc on Au(111). (**f**) d*I*/d*V* spectra of CoPc and dehydrogenated CoPc (d-CoPc) at different temperatures. Adapted from [68], with permission from American Association for the Advancement of Science, 2005.

A well-known case of spin-state manipulation via tip-induced chemical reactions was reported by Zhao et al. [68], which involves the CoPc/Au(111) system. For an intact CoPc molecule absorbed on Au(111), the d*I*/d*V* measurement showed no spin-associated signature (black curve in Figure 4f), indicating that the unpaired electron of the Co center in free CoPc was completely quenched upon adsorption due to the molecule–substrate interaction. However, the localized spin was recovered in the dehydrogenated CoPc (d-CoPc) which was generated by the sequential tip pulses at 3.6 V on top of the four indole rings (Figure 4a–e). The change in the spin state of d-CoPc was evidenced by the presence of an intense peak around the Fermi level in the d*I*/d*V* spectra which was assigned as a Kondo resonance due to its temperature evolution (colored curves in Figure 4f). Insights into the mechanism of the conversion was obtained by the comparison between the theoretical results of CoPc/Au(111) and d-CoPc/Au(111). Geometric optimizations showed a structural distortion of d-CoPc with respect to CoPc, including a larger Co-substrate separation and a smaller distance between the end carbon atoms and the substrate of d-CoPc compared with CoPc. The chemical-conversion-induced geometric changes of CoPc were accompanied with the variations in the electronic structures. For intact CoPc, the calculated spin-polarized partial density of states (PDOS) displayed the barely polarized orbitals of the Co center, which explained the absence of a magnetic moment in the intact CoPc absorbed on Au(111). As a comparison, polarized orbitals were found for d-CoPc, leading to a magnetic moment of 1.03 μ_B_, which was the origin of the Kondo resonance.

**Figure 5:**
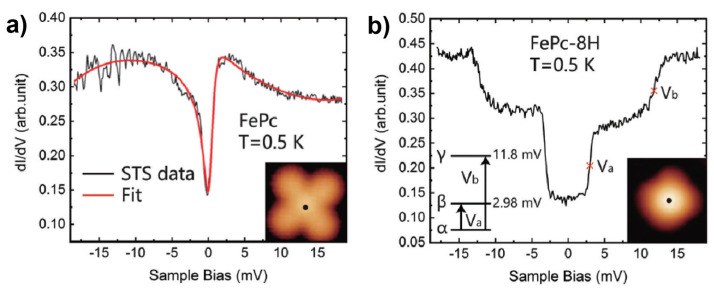
(**a**) d*I*/d*V* spectrum of intact FePc on Au(111). Inset: STM image of intact FePc. (**b**) d*I*/d*V* spectrum of dehydrogenated FePc absorbed on Au(111). Inset: Schematic diagram of the split spin states (bottom left) and STM image of dehydrogenated FePc (bottom right). Adapted from [67], with permission from the Royal Society of Chemistry, 2018.

It is worth mentioning that in addition to manipulation of spin state as presented in the case shown above, the similar tip-induced chemical reactions were also employed as an efficient approach to tuning the spin-related transport properties in another 3d-MPc system. Li et al. reported the transition from the Kondo state to the magnetic triplet state of FePc on Au(111) upon the tip-induced dehydrogenation [67]. Deprotonated FePc molecules (Figure 5b inset at the bottom right) were generated from the intact FePc molecules (Figure 5a inset) on Au(111) by tip-pulse in the similar way as that reported for the CoPc/Au(111) system [68]. d*I*/d*V* investigations uncovered the distinct spectroscopic features of the intact and dehydrogenated species. For intact FePc, the zero-bias anomaly in the d*I*/d*V* spectrum (Figure 5a) was interpreted as the Kondo resonances originated from two unpaired d-electrons of the central Fe ion screened by the substrate electron gas. Once deprotonated, the structural distortion of the molecule resulted in the increased Fe–substate separation, which led to the weakened hybridization between the localized d-electrons of Fe and the delocalized electron gas of the Au substrate. As a consequence, the Kondo features were replaced by a double-step feature in the d*I*/d*V* spectrum (Figure 5b), which indicated the emergence of inelastic electron tunneling. These inelastic electron tunneling steps reflected the spin transitions between the α, β and γ states (Figure 5b inset at the bottom left) that were originated from the splitting of the *S* = 1 state of FePc due to the spin–orbit interaction.

**Figure 6:**
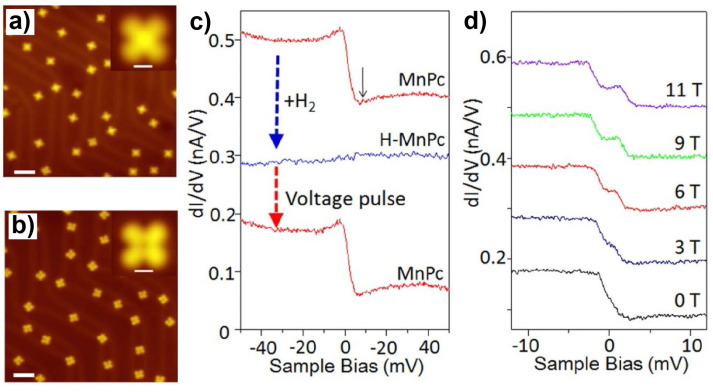
STM images of (**a**) MnPc and (**b**) H-MnPc on Au(111). (**c**) Sequential variations of d*I*/d*V* spectra recorded at the center of a MnPc molecule induced by the adsorption and desorption of a H atom. (**d**) Magnetic-field evolution of the Kondo feature of MnPc. Adapted from [64], with permission from Macmillan Publishers Limited, 2013.

Due to the irreversibility of the tip-induced chemical conversions, the above-described strategy usually shows a one-way effect to the molecular spin systems. As another frequently used strategy, the chemical adsorption of atoms or small molecules provides a reversible approach to tuning the molecular spins. One example involving the MnPc/Au(111) system was reported by Liu and coworkers [64]. MnPc molecules absorbed on Au(111) exhibited a protruding cross feature (Figure 6a). H_2_ dosage caused the conversion of the original MnPc molecules into the structures with a depression at the center (Figure 6b). This variation in the molecular morphology induced by H_2_ dosage was attributed to the chemical adsorption of an atomic H at the Mn ion in MnPc molecules which gave rise to H-MnPc. Furthermore, it was found that the MnPc state could be recovered from the H-MnPc state by applying a positive tip-pulse at H-MnPc molecules which led to the detachment of H. The chemisorption of atomic H to MnPc also gave rise to remarkable changes in the d*I*/d*V* measurements. The d*I*/d*V* spetra of MnPc on Au(111) showed a step-shaped feature at zero bias (red curves in Figure 6c). This zero-bias anomaly was attributed to Kondo resonance according to its splitting in the presence of a magnetic field (Figure 6d). In contrast, featureless d*I*/d*V* curves were found for H-MnPc (blue curve in Figure 6c). Reversible conversions of the d*I*/d*V* spectra between the Kondo-featured and featureless curves were achieved by controlled H_2_ dosages which led to a MnPc to H-MnPc conversion and tip-pulses which resulted in the H detachment of H-MnPc (Figure 6c). Further theoretical investigations demonstrated an *S* = 3/2 state of MnPc on Au(111). The exchange interaction between the localized spin of the Mn ion and the substrate electron gas gave rise to the experimentally observed Kondo resonance. As for H-MnPc, the calculations unraveled an almost unchanged number of d-electrons of the Mn ion but redistributed effective charges in d orbitals compared with MnPc. The latter was responsible for the reduction in the net spin of the molecule from *S* = 3/2 for MnPc to *S* = 1 for H-MnPc. Moreover, H attachment also resulted in a larger Mn–substrate separation and thus weakened Mn–substrate coupling for H-MnPc. Both factors contributed to the suppression of the Kondo effect in H-MnPc. In all, the chemisorption of atomic H served as an efficient approach to tuning the spin state of MnPc on Au(111) reversibly. The similar strategy was also employed for the manipulation of the spin state of MnPc on Bi(110) between *S* = 1 and *S* = 1/2 states where CO instead of atomic H was used as the adsorbate [65].

### 3.2. Spin Crossover (SCO) Complexes

Spin crossover (SCO) complexes are a family of coordination compounds featuring switchable spin-state bistability in which transition metals with 3d*^n^* (*n* = 4–7) electronic configuration serve as the spin centers. Upon external stimuli such as temperature, light irradiation, pressure, or electric field [70,71,72,73], the metal centers of SCO molecules can switch between the low-spin (LS) state and high-spin (HS) state. The origin of the spin-state switching lies in the magneto-structural effect of SCO complexes, that is, the change in coordination geometry can give rise to rearrangement of d orbitals and hence a variation in the spin state of the metal center. 

Investigations of SCO phenomena of individual molecules at molecular scale have been widely carried out by STM. One recent example was reported by Kobke et al. [74] showing that the spin state of Ni in a series of Ni–porphyrin complexes could switch between a LS (*S* = 0) state and a HS (*S* = 1) state through reversibly changing the molecular structure between the ruffled and flat conformations. The two different molecular conformations were stabilized by the coordination and non-coordination of the central Ni with an axial ligand, respectively. A number of other cases have been overviewed in a recent review [75].

**Figure 7:**
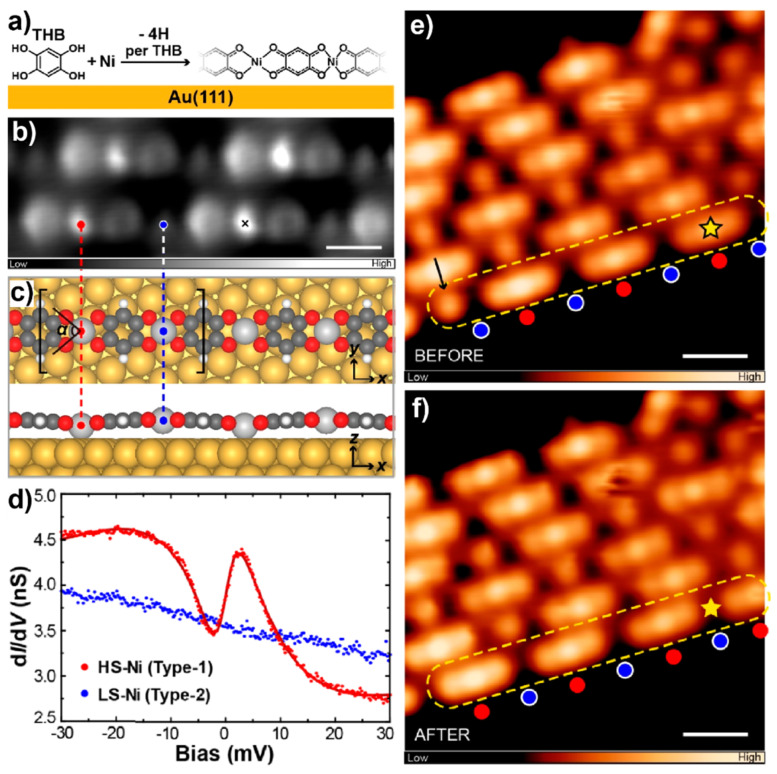
(**a**) Schematic illustration of the coordination reaction between tetrahydroxybenzene (THB) and Ni on Au(111). (**b**) STM image of the coordination chains. (**c**) Optimized models of the coordination chain. (**d**) d*I*/d*V* spectra acquired at a HS-Ni and a LS-Ni, respectively. STM images of the coordination chains (**e**) before and (**f**) after the tip-induced collective switching within the marked chain. Adapted from [78], with permission from American Chemical Society, 2020.

Once assembled into extended structures, SCO materials may emerge various spin-state orderings and exhibit a rich variety of collective dynamic behaviors [76]. However, in contrast to the extensively explored individual SCO systems, only a few molecular-level studies have focused on the SCO phenomena in ensembles [77,78]. In a recent work by Liu et al. [78], chemically bonded one-dimensional SCO chains that were constructed via coordination between Ni and deprotonated tetrahydroxybenzene (THB) molecules on Au(111) (Figure 7a) were scrutinized by STM. STM imaging resolved the alternate arrangement of two types of Ni atoms, i.e., the bright ones and the dark ones, along the chains (Figure 7b). Theoretical optimization of the coordination structure (Figure 7c) demonstrated different coordination geometries of the two types of Ni atoms: longer (shorter) Ni–O bonds, smaller (larger) O–Ni–O angles, and a smaller (larger) Ni–substrate separation for the bright (dark) Ni. Different spin states of the two types of Ni atoms were identified by combined d*I*/d*V* measurements and theoretical calculations. The dark Ni atoms exhibited featureless d*I*/d*V* spectra near the Fermi level (blue curve in Figure 7d). This experimental observation was in accordance with the calculation result which showed barely polarized d orbitals of the dark Ni atoms, indicating their *S* = 0 (LS) state. As a comparison, zero-bias anomaly was found for the bright Ni atoms (red curve in Figure 7d) and was attributed to Kondo resonances according to the magnetic-field evolution. Theoretical results revealed the highly polarized d orbitals of the bright Ni atoms whose interaction with the substrate electron gas was responsible for the Kondo features in the d*I*/d*V* spectra. A magnetic moment of 1.59 μ_B_ was concluded for the bright Ni atoms, corresponding to an *S* = 1 (HS) state. As a consequence, the Ni atoms in the coordination chains were at a HS (*S* = 1) or a LS (*S* = 0) state alternately along the chains, giving rise to a unique spin-state ordering known as the antiferroelastic phase. With a magneto-structural dependence, the Ni atoms in the chains could play as the SCO centers when being excited by the tip-pulse located upon. Interestingly, it was found that such SCO behavior, i.e., the spin-state switching between HS and LS states, of multiple Ni atoms in the same coordination chain could take place collectively (Figure 7e,f) upon the tip excitation of a single Ni atom (marked by the stars in Figure 7e,f). Such collective conversions actually resulted in the reversible switching of the chain between two degenerate antiferroelastic states, that is, the transition between the spin-state configurations of “...101010...” and “...010101...”. A domino-like dynamic magneto-structural relaxation process was proposed to explain the mechanism of the collective SCO phenomenon. 

## 4. Spin State Manipulation of Molecules Comprising 4f-Metals

Lanthanide (Ln) Pc molecules, e.g., double-decker (LnPc_2_) and triple-decker (Ln_2_Pc_3_) complexes, are a promising class of single-molecule magnets (SMMs) [79]. Their large magnetic moments are originated from the 4f electron states of the Ln ion. In the recent decade, STM has been extensively employed for exploring the on-surface systems of Ln–Pc complexes, especially the relatively stable LnPc_2_ species, focusing on their self-assembly, electronic structures, magnetic properties, and so on [80,81,82,83,84,85,86,87,88,89,90,91,92,93,94]. However, due to the inner-core nature of the 4f orbitals which leads to the little contribution of the 4f electrons to the tunneling current, the direct detection of the 4f electron states are elusive by STM measurement in some surface-confined LnPc_2_ systems [83,85,89,93]. In fact, there remain substantial debates on whether the spin states originated from the 4f electrons in LnPc_2_ molecules can be directly visualized by STM [81,83,85,86,89,91,94]. Nevertheless, the spin state manipulation has been achieved in several LnPc_2_ systems by tuning the unpaired π-electrons residing on the Pc ligands [83,93].

**Figure 8:**
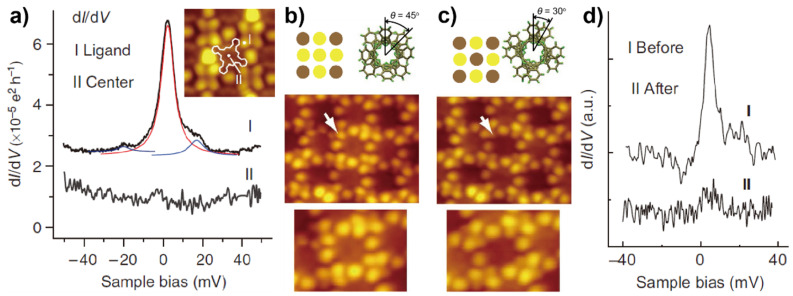
(**a**) d*I*/d*V* spectra recorded at the lobe and center of a TbPc_2_ molecule on Au(111). Inset: STM image of TbPc_2_ molecules in the assembled structure. Schematic illustrations and STM images of TbPc_2_ molecules with (**b**) *θ* = 45° and (**c**) *θ* = 30°. (**d**) d*I*/d*V* spectra acquired at a TbPc_2_ molecule before and after the application of a tip pulse. Adapted from [83], with permission from Macmillan Publishers Limited, 2011.

Komeda and coworkers reported the switch-on and off of a π-electron spin of TbPc_2_ on Au(111) by rotating the upper Pc ligand with controlled current pulses [83]. There are two spin systems in TbPc_2_, that is, the Tb ion and an unpaired electron in the ligand π orbital. Although the magnetic signature of the former was invisible by STM in this case, the Kondo effect of the latter was captured by spectroscopic investigations. The d*I*/d*V* measurement revealed the site dependence of the Kondo feature: the Kondo peak was prominent around the lobes of the molecule but was dramatically attenuated at the center (Figure 8a). This served as an evidence showing that it was the spin residing on the Pc ligand that was responsible for the Kondo resonance. Then, it was found that the Kondo feature could be switched on and off when the upper Pc ligand was rotated by applying a tip pulse to the molecule. For a TbPc_2_ molecule with an azimuthal rotational angle (*θ*) between the two Pc ligands of 45° whose center appeared brighter in the STM images (Figure 8b), the Kondo peak could be detected (top curve in Figure 8d). Once pulsed, the center of the molecule became darker, which indicated a change in *θ* of the molecule to 30° (Figure 8c). Meanwhile, the d*I*/d*V* measurement showed the disappearance of the Kondo resonance for the pulsed molecules (bottom curve in Figure 8d). This *θ*-dependent switching of the Kondo effect was interpreted as a result of the variation in the molecular spin state. For the TbPc_2_ molecules with *θ* = 45°, the Pc ligand of the molecule absorbed on Au(111) had an *S* = 1/2 spin originated from the unpaired π-electron. However, the rotation of the upper ligand to *θ* = 30° led to the rearrangement of the frontier molecular orbitals, which resulted in the charge transfer from the surface, therefore quenching the molecular spin and hence the Kondo state. 

**Figure 9:**
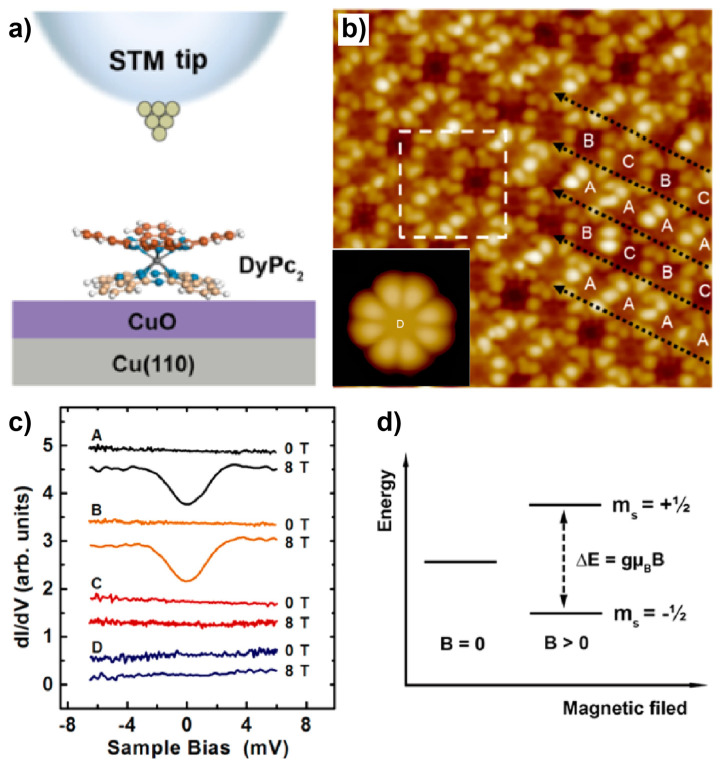
(**a**) Schematic illustration of the double-barrier tunneling junction (DBTJ) composed of STM tip, vacuum, DyPc_2_ molecule, CuO film, and Cu(110) substrate. (**b**) STM image of the molecules A, B, and C in the assembled structure and the isolated molecule D (inset). (**c**) d*I*/d*V* spectra of molecules A, B, C, and D under magnetic fields of *B* = 0 T and 8 T. (**d**) Schematic showing the Zeeman splitting. Adapted from [93], with permission from American Chemical Society, 2018.

The investigation of DyPc_2_ in the double-barrier tunneling junction (DBTJ) consisting of vacuum and CuO film (Figure 9a) by Zhang et al. [93] is also inspiring for the manipulation of the ligand spin of LnPc_2_ molecules. The adsorption of DyPc_2_ on the ultrathin CuO film on Cu(110) led to four types of molecules, that is A, B, and C in the assembled structures (Figure 9b) and the isolated molecules D (Figure 9b inset). Similar to TbPc_2_, DyPc_2_ also possesses two spin systems in its neutral state, i.e., the Dy ion whose 4f electrons are undetectable by STM measurement, and the unpaired π-electron delocalized on the Pc ligands. Spectroscopic studies were carried out for characterizing the ligand spin. Molecules A and B showed featureless d*I*/d*V* curves under zero magnetic field but a remarkable dip at the Fermi level under a magnetic field of 8 T (black and orange curves in Figure 9c). The emergence of the zero-bias feature under the nonzero external magnetic field (*B*) was attributed to the spin excitation of the molecule from *m*_s_ = −1/2 to *m*_s_ = +1/2 which was originated from the Zeeman splitting of the degenerated *m*_s_ = ±1/2 spin states at *B* = 8 T (Figure 9d). Therefore, it was concluded that molecules A and B possessed one unpaired π-electron, indicating that they were in neutral states. In contrast, the featureless spectra were found for molecules C and D at both *B* = 0 and 8 T (red and blue curves in Figure 9c), meaning that their unpaired π-electrons were quenched and thus they were charged. Given the dependence between the spin state and charge state of DyPc_2_ in the DBTJ, the manipulation of the charge state could hence lead to the change in the spin state. It was demonstrated that the charge state of DyPc_2_ in this system could be tuned by applying different biases of the DBTJ, which would give rise to the switching-on and off of the ligand spin of DyPc_2_.

## 5. Conclusions

In this review, we summarize the recent investigations of spin state manipulation of surface-confined molecules in three categories, i.e., molecules composed of main group elements such as verdazyl radicals, ReA and AlPc, molecules comprising 3d-metals such as 3d-MPcs and the spin crossover coordination chains, and molecules comprising 4f-metals such as TbPc_2_ and DyPc_2_. Examples are presented to show the efficient effect of several spin state tuning strategies, including chemical reactions, reversible atomic/molecular chemisorption, and STM-tip manipulations, in different on-surface molecular systems. The employment of STM and STS in these studies enables the exploration of the spin-related phenomena and properties at molecular level by detecting the Kondo effect or spin excitation of the molecular systems. These case studies contribute to the fundamental understanding of molecular magnetism and spintronics.

To make further steps toward the practical application of the magnetic molecules as spintronic and quantum computing devices, the studies focusing on the magnetic molecular systems located on carbon-based substrates or nanostructures are of great significance. On the one hand, the work by Cervetti et al. concerning the molecular-spin–graphene hybrids has demonstrated that the interaction between magnetic molecules and graphene has an essential influence on the quantum dynamics of molecular spins [95]. Further explorations in the field have involved a variety of coupled systems formed between different molecular spins and graphene, aiming at elucidating their structures, electronic properties, magnetism, and so forth [61,96,97,98,99,100,101]. On the other hand, the hybrid systems composed of single molecular magnets and carbon nanotubes (CNTs) also have drawn extensive attention [102,103,104,105]. A strong spin–phonon coupling between molecular spins and CNTs was demonstrated, which can enhance the sensitivity of CNT-based magnetometers and ultimately enable coherent spin manipulation and quantum entanglement [106,107]. Such hybrid systems were also found to be able to play as spintronic devices such as spin valves [108]. More about the research progress focusing on the hybrids consisting of molecular magnets and carbon-based materials can be found in the reviews by Cervetti et al. [109] and Pineda et al. [110], just to name a few. Based on these excellent previous works, the systematic investigation by STM of spin manipulation of magnetic molecules coupled with carbon-based substrates is expected to provide more fundamental insights into the hybrid systems at molecular level, which should be inspiring for the design of molecule-based spintronic and quantum computing devices.

## References

[1] Chappert C., Fert A., Dau F.N.V. (2007). The emergence of spin electronics in data storage. Nat. Mater..

[2] Gatteschi D., Bogani L., Cornia A., Mannini M., Sorace L., Sessoli R. (2008). Molecular magnetism, status and perspectives. Solid State Sci..

[3] Robertsona N., Yee G.T., Bruce D.W., O’Hare D., Walton R.I. (2010). Molecular magnetic materials. Molecular Materials.

[4] Coronado E. (2019). Molecular magnetism: From chemical design to spin control in molecules, materials and devices. Nat. Rev. Mater..

[5] Bogani L., Wernsdorfer W. (2008). Molecular spintronics using single-molecule magnets. Nat. Mater..

[6] Sanvito S. (2011). Molecular spintronics. Chem. Soc. Rev..

[7] Guo L., Gu X., Zhu X., Sun X. (2019). Recent advances in molecular spintronics: Multifunctional spintronic devices. Adv. Mater..

[8] Gaita-Arino A., Luis F., Hill S., Coronado E. (2019). Molecular spins for quantum computation. Nat. Chem..

[9] Lehmann J., Gaita-Arino A., Coronadoc E., Loss D. (2009). Quantum computing with molecular spin systems. J. Mater. Chem..

[10] Camarero J., Coronado E. (2009). Molecular vs. inorganic spintronics: The role of molecular materials and single molecules. J. Mater. Chem..

[11] Wang F., Vardeny Z.V. (2009). Organic spin valves: The first organic spintronics devices. J. Mater. Chem..

[12] Affronte M. (2009). Molecular nanomagnets for information technologies. J. Mater. Chem..

[13] Timco G.A., Faust T.B., Tunaa F., Winpenny R.E.P. (2011). Linking heterometallic rings for quantum information processing and amusement. Chem. Soc. Rev..

[14] Troiania F., Affronte M. (2011). Molecular spins for quantum information technologies. Chem. Soc. Rev..

[15] Hla S.-W., Rieder K.-H. (2003). STM control of chemical reactions: Single-molecule synthesis. Annu. Rev. Phys. Chem..

[16] De Feyter S., De Schryver F.C. (2003). Two-dimensional supramolecular self-assembly probed by scanning tunneling microscopy. Chem. Soc. Rev..

[17] Barth J.V. (2007). Molecular architectonic on metal surfaces. Annu. Rev. Phys. Chem..

[18] Zhang J.L., Zhong J.Q., Lin J.D., Hu W.P., Wu K., Xu G.Q., Wee A.T., Chen W. (2015). Towards single molecule switches. Chem. Soc. Rev..

[19] Ko W., Ma C., Nguyen G.D., Kolmer M., Li A.P. (2019). Atomic-scale manipulation and in situ characterization with scanning tunneling microscopy. Adv. Funct. Mater..

[20] Chen X., Fu Y.S., Ji S.H., Zhang T., Cheng P., Ma X.C., Zou X.L., Duan W.H., Jia J.F., Xue Q.K. (2008). Probing superexchange interaction in molecular magnets by spin-flip spectroscopy and microscopy. Phys. Rev. Lett..

[21] Tsukahara N., Noto K., Ohara M., Shiraki S., Takagi N., Takata Y., Miyawaki J., Taguchi M., Chainani A., Shin S. (2009). Adsorption-induced switching of magnetic anisotropy in a single iron(II) phthalocyanine molecule on an oxidized Cu(110) surface. Phys. Rev. Lett..

[22] Wegner D., Yamachika R., Zhang X., Wang Y., Baruah T., Pederson M.R., Bartlett B.M., Long J.R., Crommie M.F. (2009). Tuning molecule-mediated spin coupling in bottom-up-fabricated vanadium-tetracyanoethylene nanostructures. Phys. Rev. Lett..

[23] Tsukahara N., Shiraki S., Itou S., Ohta N., Takagi N., Kawai M. (2011). Evolution of Kondo resonance from a single impurity molecule to the two-dimensional lattice. Phys. Rev. Lett..

[24] Dilullo A., Chang S.H., Baadji N., Clark K., Klockner J.P., Prosenc M.H., Sanvito S., Wiesendanger R., Hoffmann G., Hla S.W. (2012). Molecular Kondo chain. Nano Lett..

[25] Kahle S., Deng Z., Malinowski N., Tonnoir C., Forment-Aliaga A., Thontasen N., Rinke G., Le D., Turkowski V., Rahman T.S. (2012). The quantum magnetism of individual manganese-12-acetate molecular magnets anchored at surfaces. Nano Lett..

[26] Minamitani E., Tsukahara N., Matsunaka D., Kim Y., Takagi N., Kawai M. (2012). Symmetry-driven novel Kondo effect in a molecule. Phys. Rev. Lett..

[27] Burgess J.A., Malavolti L., Lanzilotto V., Mannini M., Yan S., Ninova S., Totti F., Rolf-Pissarczyk S., Cornia A., Sessoli R. (2015). Magnetic fingerprint of individual Fe_4_ molecular magnets under compression by a scanning tunnelling microscope. Nat. Commun..

[28] Hiraoka R., Minamitani E., Arafune R., Tsukahara N., Watanabe S., Kawai M., Takagi N. (2017). Single-molecule quantum dot as a Kondo simulator. Nat. Commun..

[29] Huang Z., Zhang Y., He Y., Song H., Yin C., Wu K. (2017). A chemist’s overview of surface electron spins. Chem. Soc. Rev..

[30] Choi T., Badal M., Loth S., Yoo J.-W., Lutz C.P., Heinrich A.J., Epstein A.J., Stroud D.G., Gupta J.A. (2014). Magnetism in single metalloorganic complexes formed by atom manipulation. Nano Lett..

[31] Ternes M., Heinrich A.J., Schneider W.D. (2009). Spectroscopic manifestations of the Kondo effect on single adatoms. J. Phys. Condens. Matter..

[32] Heinrich A.J., Gupta J.A., Lutz C.P., Eigler D.M. (2004). Single-atom spin-flip spectroscopy. Science.

[33] Ternes M. (2015). Spin excitations and correlations in scanning tunneling spectroscopy. New J. Phys..

[34] Requist R., Modesti S., Pier Paolo Baruselli P.P., Smogunov A., Fabrizio M., Tosatti E. (2014). Kondo conductance across the smallest spin 1/2 radical molecule. Proc. Natl. Acad. Sci. USA.

[35] Mishra S., Lohr T.G., Pignedoli C.A., Liu J., Berger R., Urgel J.I., Müllen K., Feng X., Ruffieux P., Fasel R. (2018). Tailoring bond topologies in open-shell graphene nanostructures. ACS Nano.

[36] Liu J., Mishra S., Pignedoli C.A., Daniele Passerone D., Urgel J.I., Fabrizio A., Lohr T.G., Ma J., Komber H., Baumgarten M. (2019). Open-shell nonbenzenoid nanographenes containing two pairs of pentagonal and heptagonal rings. J. Am. Chem. Soc..

[37] Lombardi F., Lodi A., Ma J., Liu J., Slota M., Narita A., Myers W.K., Müllen K., Feng X., Bogani L. (2019). Quantum units from the topological engineering of molecular graphenoids. Science.

[38] Mishra S., Beyer D., Eimre K., Liu J., Berger R., Groning O., Pignedoli C.A., Mullen K., Fasel R., Feng X. (2019). Synthesis and characterization of π-extended triangulene. J. Am. Chem. Soc..

[39] Mishra S., Beyer D., Eimre K., Kezilebieke S., Berger R., Gröning O., Pignedoli C.A., Müllen K., Peter Liljeroth P., Ruffieux P. (2020). Topological frustration induces unconventional magnetism in a nanographene. Nat. Nanotechnol..

[40] Sun Q., Yao X., Gröning O., Eimre K., Pignedoli C.A., Müllen K., Narita A., Fasel R., Ruffieux P. (2020). Coupled spin states in armchair graphene nanoribbons with asymmetric zigzag edge extensions. Nano Lett..

[41] Mishra S., Beyer D., Eimre K., Ortiz R., Fernandez-Rossier J., Berger R., Groning O., Pignedoli C.A., Fasel R., Feng X. (2020). Collective all-carbon magnetism in triangulene dimers. Angew. Chem. Int. Ed..

[42] Sun Q., Mateo L.M., Robles R., Ruffieux P., Lorente N., Bottari G., Torres T., Fasel R. (2020). Inducing open-shell character in porphyrins through surface-assisted phenalenyl π-extension. J. Am. Chem. Soc..

[43] Mishra S., Beyer D., Berger R., Liu J., Groning O., Urgel J.I., Mullen K., Ruffieux P., Feng X., Fasel R. (2020). Topological defect-induced magnetism in a nanographene. J. Am. Chem. Soc..

[44] Liu J., Isshiki H., Katoh K., Morita T., Breedlove B.K., Yamashita M., Komeda T. (2013). First observation of a Kondo resonance for a stable neutral pure organic radical, 1,3,5-triphenyl-6-oxoverdazyl, adsorbed on the Au(111) surface. J. Am. Chem. Soc..

[45] Mullegger S., Rashidi M., Fattinger M., Koch R. (2013). Surface-supported hydrocarbon pi radicals show Kondo behavior. J. Phys. Chem. C.

[46] Zhang Y.-H., Kahle S., Herden T., Stroh C., Mayor M., Schlickum U., Ternes M., Wahl P., Kern K. (2013). Temperature and magnetic field dependence of a Kondo system in the weak coupling regime. Nat. Commun..

[47] Garnica M., Stradi D., Barja S., Calleja F., Díaz C., Alcamí M., Martín N., Vázquez de Parga A.L., Martín F., Miranda R. (2013). Long-range magnetic order in a purely organic 2D layer adsorbed on epitaxial graphene. Nat. Phys..

[48] Fernandez-Torrente I., Franke K.J., Pascual J.I. (2008). Vibrational Kondo effect in pure organic charge-transfer assemblies. Phys. Rev. Lett..

[49] Simao C., Mas-Torrent M., Crivillers N., Lloveras V., Artes J.M., Gorostiza P., Veciana J., Rovira C. (2011). A robust molecular platform for non-volatile memory devices with optical and magnetic responses. Nat. Chem..

[50] Frisenda R., Gaudenzi R., Franco C., Mas-Torrent M., Rovira C., Veciana J., Alcon I., Bromley S.T., Burzurí E., van der Zant H.S.J. (2015). Kondo effect in a neutral and stable all organic radical single molecule break junction. Nano Lett..

[51] Lloveras V., Badetti E., Veciana J., Vidal-Gancedo J. (2016). Dynamics of intramolecular spin exchange interaction of a nitronyl nitroxide diradical in solution and on surfaces. Nanoscale.

[52] Gaudenzi R., Burzurí E., Reta D., Moreira I.D.P.R., Bromley S.T., Rovira C., Veciana J., van der Zant H.S.J. (2016). Exchange coupling inversion in a high-spin organic triradical molecule. Nano Lett..

[53] Karan S., Berndt R. (2016). Generation of spin in single cholesterol molecules on gold. Phys. Chem. Chem. Phys..

[54] Karan S., Li N., Zhang Y., He Y., Hong I.P., Song H., Lu J.T., Wang Y., Peng L., Wu K. (2016). Spin manipulation by creation of single-molecule radical cations. Phys. Rev. Lett..

[55] Zhang X., Li N., Zhang Y., Berndt R., Wang Y. (2017). 13-cis-retinoic acid on coinage metals: Hierarchical self-assembly and spin generation. Phys. Chem. Chem. Phys..

[56] Bocquet M.-L., Lorente N., Berndt R., Gruber M. (2019). Spin in a closed-shell organic molecule on a metal substrate generated by a Sigmatropic reaction. Angew. Chem. Int. Ed..

[57] Hong I.P., Li N., Zhang Y.J., Wang H., Song H.J., Bai M.L., Zhou X., Li J.L., Gu G.C., Zhang X. (2016). Vacuum synthesis of magnetic aluminum phthalocyanine on Au(111). Chem. Commun..

[58] Li Z., Li B., Yang J., Hou J. (2010). Single-molecule chemistry of metal phthalocyanine on noble metal surfaces. Acc. Chem. Res..

[59] Wang Y., Wu K., Kröger J., Berndt R. (2012). Structures of phthalocyanine molecules on surfaces studied by STM. AIP Adv..

[60] Malavolti L., Briganti M., Hanze M., Serrano G., Cimatti I., McMurtrie G., Otero E., Ohresser P., Totti F., Mannini M. (2018). Tunable spin−superconductor coupling of spin 1/2 vanadyl phthalocyanine molecules. Nano Lett..

[61] Cimatti I., Bondi L., Serrano G., Malavolti L., Cortigiani B., Velez-Fort E., Betto D., Ouerghi A., Brookes N.B., Loth S. (2019). Vanadyl phthalocyanines on graphene/SiC(0001): Toward a hybrid architecture for molecular spin qubits. Nanoscale Horiz..

[62] Franke K.J., Schulze G., Pascual J.I. (2011). Competition of superconducting phenomena and Kondo screening at the nanoscale. Science.

[63] Fu Y.S., Ji S.H., Chen X., Ma X.C., Wu R., Wang C.C., Duan W.H., Qiu X.H., Sun B., Zhang P. (2007). Manipulating the Kondo resonance through quantum size effects. Phys. Rev. Lett..

[64] Liu L., Yang K., Jiang Y., Song B., Xiao W., Li L., Zhou H., Wang Y., Du S., Ouyang M. (2013). Reversible single spin control of individual magnetic molecule by hydrogen atom adsorption. Sci. Rep..

[65] Strozecka A., Soriano M., Pascual J.I., Palacios J.J. (2012). Reversible change of the spin state in a manganese phthalocyanine by coordination of CO molecule. Phys. Rev. Lett..

[66] Gao L., Ji W., Hu Y.B., Cheng Z.H., Deng Z.T., Liu Q., Jiang N., Lin X., Guo W., Du S.X. (2007). Site-specific Kondo effect at ambient temperatures in iron-based molecules. Phys. Rev. Lett..

[67] Li R., Li N., Wang H., Weismann A., Zhang Y., Hou S., Wu K., Wang Y. (2018). Tuning the spin-related transport properties of FePc on Au(111) through single-molecule chemistry. Chem. Commun..

[68] Zhao A., Li Q., Chen L., Xiang H., Wang W., Pan S., Wang B., Xiao X., Yang J., Hou J.G. (2005). Controlling the Kondo effect of an adsorbed magnetic ion through its chemical bonding. Science.

[69] Mugarza A., Krull C., Robles R., Stepanow S., Ceballos G., Gambardella P. (2011). Spin coupling and relaxation inside molecule-metal contacts. Nat. Commun..

[70] Gutlich P., Hauser A., Spiering H. (1994). Thermal and optical switching of iron(II) complexes. Angew. Chem. Int. Ed. Engl..

[71] Marchivie M., Guionneau P., Howard J.A.K., Chastanet G., Létard J.-F., Goeta A.E., Chasseau D. (2002). Structural characterization of a photoinduced molecular switch. J. Am. Chem. Soc..

[72] Baadji N., Piacenza M., Tugsuz T., Della Sala F., Maruccio G., Sanvito S. (2009). Electrostatic spin crossover effect in polar magnetic molecules. Nat. Mater..

[73] Wang Y., Zhou Z., Wen T., Zhou Y., Li N., Han F., Xiao Y., Chow P., Sun J., Pravica M. (2016). Pressure-driven cooperative spin-crossover, large-volume collapse, and semiconductor-to-metal transition in manganese(II) honeycomb lattices. J. Am. Chem. Soc..

[74] Kobke A., Gutzeit F., Rohricht F., Schlimm A., Grunwald J., Tuczek F., Studniarek M., Longo D., Choueikani F., Otero E. (2020). Reversible coordination-induced spin-state switching in complexes on metal surfaces. Nat. Nanotechnol..

[75] Kumar K.S., Ruben M. (2020). Sublimable spin crossover complexes: From spin-state switching to molecular devices. Angew. Chem. Int. Ed..

[76] Paez-Espejo M., Sy M., Boukheddaden K. (2016). Elastic frustration causing two-step and multistep transitions in spin-crossover solids: Emergence of complex antiferroelastic structures. J. Am. Chem. Soc..

[77] Bairagi K., Iasco O., Bellec A., Kartsev A., Li D., Lagoute J., Chacon C., Girard Y., Rousset S., Miserque F. (2016). Molecular-scale dynamics of light-induced spin cross-over in a two-dimensional layer. Nat. Commun..

[78] Liu J., Gao Y., Wang T., Xue Q., Hua M., Wang Y., Huang L., Lin N. (2020). Collective spin manipulation in antiferroelastic spin-crossover metallo-supramolecular chains. ACS Nano.

[79] Woodruff D.N., Winpenny R.E., Layfield R.A. (2013). Lanthanide single-molecule magnets. Chem. Rev..

[80] Gomez-Segura J., Diez-Perez I., Ishikawa N., Nakano M., Veciana J., Ruiz-Molina D. (2006). 2-D self-assembly of the bis(phthalocyaninato)terbium(III) single-molecule magnet studied by scanning tunnelling microscopy. Chem. Commun..

[81] Vitali L., Fabris S., Conte A.M., Brink S., Ruben M., Baroni S., Kern K. (2008). Electronic structure of surface-supported bis(phthalocyaninato) terbium(III) single molecular magnets. Nano Lett..

[82] Katoh K., Yoshida Y., Yamashita M., Miyasaka H., Breedlove B.K., Kajiwara T., Takaishi S., Ishikawa N., Isshiki H., Zhang Y.F. (2009). Direct observation of lanthanide(III)-phthalocyanine molecules on Au(111) by using scanning tunneling microscopy and scanning tunneling spectroscopy and thin-film field-effect transistor properties of Tb(III)- and Dy(III)-phthalocyanine molecules. J. Am. Chem. Soc..

[83] Komeda T., Isshiki H., Liu J., Zhang Y.F., Lorente N., Katoh K., Breedlove B.K., Yamashita M. (2011). Observation and electric current control of a local spin in a single-molecule magnet. Nat. Commun..

[84] Fu Y.S., Schwobel J., Hla S.W., Dilullo A., Hoffmann G., Klyatskaya S., Ruben M., Wiesendanger R. (2012). Reversible chiral switching of bis(phthalocyaninato) terbium(III) on a metal surface. Nano Lett..

[85] Schwobel J., Fu Y., Brede J., Dilullo A., Hoffmann G., Klyatskaya S., Ruben M., Wiesendanger R. (2012). Real-space observation of spin-split molecular orbitals of adsorbed single-molecule magnets. Nat. Commun..

[86] Fahrendorf S., Atodiresei N., Besson C., Caciuc V., Matthes F., Blugel S., Kogerler P., Burgler D.E., Schneider C.M. (2013). Accessing 4f-states in single-molecule spintronics. Nat. Commun..

[87] He Y., Zhang Y., Hong I.P., Cheng F., Zhou X., Shen Q., Li J., Wang Y., Jiang J., Wu K. (2014). Low-temperature scanning tunneling microscopy study of double-decker DyPc_2_ on Pb surface. Nanoscale.

[88] Komeda T., Isshiki H., Liu J., Katoh K., Yamashita M. (2014). Variation of Kondo temperature induced by molecule-substrate decoupling in film formation of bis(phthalocyaninato)terbium(III) molecules on Au(111). ACS Nano.

[89] Zhang Y., Liao P., Kan J., Yin C., Li N., Liu J., Chen Q., Wang Y., Chen W., Xu G.Q. (2015). Low-temperature scanning tunneling microscopy study on the electronic properties of a double-decker DyPc_2_ molecule at the surface. Phys. Chem. Chem. Phys..

[90] Serrano G., Wiespointner-Baumgarthuber S., Tebi S., Klyatskaya S., Ruben M., Koch R., Mullegger S. (2016). Bilayer of terbium double-decker single-molecule magnets. J. Phys. Chem. C.

[91] Warner B., El Hallak F., Atodiresei N., Seibt P., Pruser H., Caciuc V., Waters M., Fisher A.J., Blugel S., van Slageren J. (2016). Sub-molecular modulation of a 4f driven Kondo resonance by surface-induced asymmetry. Nat. Commun..

[92] Amokrane A., Klyatskaya S., Boero M., Ruben M., Bucher J.P. (2017). Role of pi-radicals in the spin connectivity of clusters and networks of Tb double-decker single molecule magnets. ACS Nano.

[93] Zhang Y., Wang Y., Liao P., Wang K., Huang Z., Liu J., Chen Q., Jiang J., Wu K. (2018). Detection and manipulation of charge states for double-decker DyPc_2_ molecules on ultrathin CuO films. ACS Nano.

[94] Barhoumi R., Amokrane A., Klyatskay S., Boero M., Ruben M., Bucher J.-R. (2019). Screening the 4f-electron spin of TbPc_2_ singlemolecule magnets on metal substrates by ligand channeling. Nanoscale.

[95] Cervetti C., Rettori A., Pini M.G., Cornia A., Ana Repollés A., Luis F., Dressel M., Rauschenbach S., Kern K., Burghard M. (2016). The classical and quantum dynamics of molecular spins on graphene. Nat. Mater..

[96] Gragnaniello L., Paschke F., Erler P., Schmitt P., Barth N., Simon S., Brune H., Rusponi S., Fonin M. (2017). Uniaxial 2D superlattice of Fe_4_ molecular magnets on graphene. Nano Lett..

[97] Zheng Y., Huang L., Zhang Z., Jiang J., Wang K., Peng L.-M., Yu G. (2017). Sensitivity enhancement of graphene Hall sensors modified by single-molecule magnets at room temperature. RSC Adv..

[98] Serrano G., Velez-Fort E., Cimatti I., Cortigiani B., Malavolti L., Betto D., Ouerghi A., Brookes N.B., Mannini M., Sessoli R. (2018). Magnetic bistability of a TbPc_2_ submonolayer on a graphene/SiC(0001) conductive electrode. Nanoscale.

[99] Paschke F., Erler P., Enenkel V., Gragnaniello L., Fonin M. (2019). Bulk-like magnetic signature of individual. Fe_4_H molecular magnets on graphene. ACS Nano.

[100] Gajarushi A.S., Wasim M., Nabi R., Kancharlapalli S., Rao V.R., Rajaraman G., Subramaniam C., Shanmugam M. (2019). Lanthanide complexes as molecular dopants for realizing air-stable n-type graphene logic inverters with symmetric transconductance. Mater. Horiz..

[101] Sakurai M., Koley P., Aono M. (2019). Tunable magnetism of organometallic nanoclusters by graphene oxide on-surface chemistry. Sci. Rep..

[102] Bogani L., Danieli C., Biavardi E., Bendiab N., Barra A.-L., Dalcanale E., Wernsdorfer W., Cornia A. (2009). Single-molecule-magnet carbon-nanotube hybrids. Angew. Chem. Int. Ed..

[103] Nakanishi R., Satoh J., Katoh K., Zhang H., Breedlove B.K., Nishijima M., Nakanishi Y., Omachi H., Shinohara H., Yamashita M. (2018). DySc_2_N@C_80_ single-molecule magnetic metallofullerene encapsulated in a single-walled carbon nanotube. J. Am. Chem. Soc..

[104] Giusti A., Charron G., Mazerat S., Compain J.-D., Mialane P., Dolbecq A., Riviere E., Wernsdorfer W., Biboum R.N., Keita B. (2009). Magnetic bistability of individual single-molecule magnets grafted on single-wall carbon nanotubes. Angew. Chem. Int. Ed..

[105] Kyatskaya S., Mascaros J.R.G., Bogani L., Hennrich F., Kappes M., Wernsdorfer W., Ruben M. (2009). Anchoring of rare-earth-based single-molecule magnets on single-walled carbon nanotubes. J. Am. Chem. Soc..

[106] Ganzhorn M., Klyatskaya S., Ruben M., Wernsdorfer W. (2013). Strong spin–phonon coupling between a single-molecule magnet and a carbon nanotube nanoelectromechanical system. Nat. Nanotechnol..

[107] Ganzhorn M., Klyatskaya S., Ruben M., Wernsdorfer† W. (2013). Carbon nanotube nanoelectromechanical systems as magnetometers for single-molecule magnets. ACS Nano.

[108] Urdampilleta M., Klyatskaya S., Cleuziou J.-P., Ruben M., Wernsdorfer W. (2011). Supramolecular spin valves. Nat. Mater..

[109] Cervetti C., Heintze E., Bogani L. (2014). Interweaving spins with their environment: Novel inorganic nanohybrids with controllable magnetic properties. Dalton Trans..

[110] Pineda E.M., Komeda T., Katoh K., Yamashita M., Ruben M. (2016). Surface confinement of TbPc2-SMMs: Structural, electronic and magnetic properties. Dalton Trans..

